# Genetic factors associated with serum amylase in a Japanese population: combined analysis of copy-number and single-nucleotide variants

**DOI:** 10.1038/s10038-022-01111-3

**Published:** 2023-01-04

**Authors:** Zannatun Nayema, Takehiro Sato, Takayuki Kannon, Hiromasa Tsujiguchi, Kazuyoshi Hosomichi, Hiroyuki Nakamura, Atsushi Tajima

**Affiliations:** 1 Department of Bioinformatics and Genomics, Graduate School of Medical Sciences, Kanazawa University, Kanazawa, Japan; 2 Department of Hygiene and Public Health, Graduate School of Medical Sciences, Kanazawa University, Kanazawa, Japan; 3 Present Address: Department of Biomedical Data Science, Fujita Health University School of Medicine, Toyoake, Japan; 4 Present Address: Laboratory of Computational Genomics, School of Life Science, Tokyo University of Pharmacy and Life Sciences, Hachioji, Japan

**Keywords:** Genome-wide association studies, Genetic predisposition to disease

## Abstract

Amylase activity and levels in humans are heritable quantitative traits. Although many studies exist on the effects of copy-number variants (CNVs) in amylase genes (AMY) on human phenotypes, such as body mass index (BMI), the genetic factors controlling interindividual variation in amylase levels remain poorly understood. Here, we conducted a genome-wide association study (GWAS) of serum amylase levels (SAL) in 814 Japanese individuals to identify associated single-nucleotide variants (SNVs), after adjusting for non-genetic factors. Diploid copy numbers (CN) of AMY (*AMY1*, *AMY2A*, and *AMY2B*) were measured using droplet digital PCR to examine the association between each diploid CN and SAL. We further assessed the relative contribution of the GWAS-lead SNV and AMY CNVs to SAL. GWAS identified 14 significant SNVs (*p* < 5 × 10^−8^) within a linkage disequilibrium block near the AMY cluster on chromosome 1. The association analyses of AMY CNVs and SAL showed a significant association between *AMY1* diploid CN and SAL (*p* = 1.89 × 10^−19^), while no significant association with SAL was found for *AMY2A* CN (*p* = 0.54) or *AMY2B* CN (*p* = 0.15). In a joint association analysis with SAL using the GWAS-lead SNV and *AMY1* diploid CN, *AMY1* CN remained significant (*p* = 5.4 ×10^−13^), while the association of the lead SNV was marginal (*p* = 0.08). We also found no association between *AMY1* diploid CN and BMI (*p* = 0.14). Our results indicate that *AMY1* CNV is the major genetic factor for Japanese SAL, with no significant association with BMI.

## Introduction

Amylase, secreted predominantly by the salivary glands and pancreas, is one of the key enzymes that aid in the digestion of dietary starches [1]. Serum amylase consists of an approximately equal amount of the salivary and pancreatic types [1]. Elevated amylase levels are used as conventional biomarkers for acute pancreatitis [2] and indicators of psychological stress [3]. In recent years, individual differences in amylase levels, even within the normal range have attracted attention as they are significantly associated with food perception and preference [3], gut microbial patterns [4], metabolic syndrome, diabetes [5], obesity, cardiovascular diseases [6], and neurological diseases [7, 8]. However, the important determinants of individual differences in amylase levels and their pathophysiological role remain unclear.

The estimated heritability of human amylase activity in urine and plasma ranges from 0.44 to 0.53 [9]. Amylase activity and levels can be considered a heritable quantitative trait. Until recently, studies on genetic variants associated with amylase levels have been limited to copy-number variants (CNVs) in the salivary amylase genes (*AMY1A*, *AMY1B*, and *AMY1C*, collectively referred to as *AMY1*) and pancreatic amylase genes (*AMY2A* and *AMY2B*) [10]. Human amylase genes (*AMY*)map to a region of complex genomic structure with duplications, inversions, and deletions on the short arm of chromosome 1 [11]. Previous studies have reported individual variations in diploid copy numbers (CNs) of the *AMY1*, *AMY2A*, and *AMY2B* genes, with diploid CNVs ranging from 1–27 for *AMY1* [12], and 2–6 for *AMY2A* and *AMY2B* [13], respectively. Although diploid CN of *AMY1* can explain some (~10–35%) of the interindividual variations in amylase levels [14–17], the remaining unexplained phenotypic variation suggests the presence of additional genetic factors. Over the last few decades, many studies have reported consistent findings of negative correlations between amylase levels or amylase activity and obesity-related traits [6, 18], but there remains controversy regarding the association of *AMY1* CN with obesity or BMI [13, 19–21]. Elucidating the complex genetic architecture underlying the amylase levels will provide a deeper understanding of the role of amylase in susceptibility to various diseases and evolutionary adaptation in humans [12, 15, 19, 22–24].

Serum amylase levels (SALs) are also influenced by non-genetic factors such as dietary habits and lifestyle [5]. To our knowledge, no genome-wide association studies (GWASs) have been conducted to analyze genetic factors associated with SAL while adjusting for the effects of known non-genetic factors. Therefore, the purpose of this study was to evaluate genetic factors associated with interindividual variations in SAL in a Japanese cohort. To identify single-nucleotide variants (SNVs) associated with SAL, we first performed a GWAS of SAL with known non-genetic factors as covariates using genetically unrelated participants in a population-based cohort. In addition, the diploid CNs of AMY genes (*AMY1*, *AMY2A*, and *AMY2B*), which are known genetic factors contributing to individual differences in SAL, were experimentally determined in the study participants, and the association of the diploid CNs of *AMY1*, *AMY2A*, and *AMY2B* with SAL was analyzed. Subsequently, a multivariate regression analysis was performed to determine the relative contribution of GWAS-identified SNVs and amylase CNVs to SAL. We finally evaluated the association between SAL-associated genetic factors, including amylase CNVs and BMI.

## Materials and methods

### Study design and participants

This study was part of the Shikamachi health improvement practice (SHIP) genome cohort study, a longitudinal observational study of the residents of Shika Town, located in Ishikawa Prefecture, Japan, as previously described [25]. The SHIP cohort study was conducted according to the guidelines laid down in the Declaration of Helsinki and was approved by the medical ethics committee of Kanazawa University, Japan (protocol code, 1491; date of approval, December 18, 2013). All the participants gave their informed consent prior to their inclusion in the study.

This study included 1150 participants over the age of 40 years, collected between 2013 and 2017. In the baseline survey of the SHIP cohort, each participant underwent a detailed medical examination, including anthropometric measurements such as height and weight, and completed comprehensive questionnaires on diet, lifestyle, and health assessment, including age, sex, smoking status, and drinking habits.

### Serum amylase measurement, DNA extraction, and other variables

Peripheral blood samples were collected in fasting conditions for biochemical tests and DNA isolation. Serum amylase concentration (U/l) was measured by a clinical laboratory test using Et-G7-pNP (4,6-ethylidene-4-nitrophenyl-alpha-1,4-d-maltoheptaoside) as a substrate according to the Japan Society of Clinical Chemistry transferable method at SRL, Inc. (Tokyo, Japan). Genomic DNA was extracted from blood samples anticoagulated with EDTA following the manufacturer’s protocol using a QIAamp DNA Blood Maxi kit (QIAGEN Inc., Hilden, Germany) or entrusted to SRL, Inc. (Tokyo, Japan).

The 5 variables used as covariates in assessing the association between genetic factors and serum amylase level (SAL), because of their potential associations with SAL, were age, sex, body mass index (BMI), smoking status, and drinking habits [5]. Age and sex were self-reported. BMI was calculated by dividing body weight in kilograms by height in meters squared (kg/m^2^). Smoking status and drinking habits were measured as categorical variables based on the questionnaire. Smoking status was categorized as current smoker or non-current smoker. Based on alcohol consumption, drinking habits were categorized into drinkers (those who had alcoholic beverages equivalent to 60 g of ethanol or more, at least 5 days per week) or non-drinkers. Univariate linear regression analysis was performed to assess associations between SAL and the 5 covariates.

### SNV genotyping, quality control, and imputation

Genome-wide SNV genotyping was performed using the Japonica Array v2 [26] (TOSHIBA Inc., Tokyo, Japan) following the manufacturer’s instructions. Detailed procedures of quality control for the genome-wide SNV data (approximately 675,000 SNVs) and subsequent genotype imputation were as previously described [27]. In summary, we excluded 9 individuals with inconsistency between the reported sex and the karyotype. SNVs and one individual with call rates <98% were eliminated. SNVs with minor allele frequency (MAF) < 1% or significant deviation from the Hardy–Weinberg equilibrium (*p* < 0.005) were also excluded. Thirty individuals with an estimated inbreeding coefficient >0.0625 from SNV data were removed. We ensured the inclusion of only unrelated individuals by excluding 282 genetically related individuals based on the criterion of excluding one individual per pair when there was a proportion of shared identity by descent ($\hat \pi$) higher than 0.125 (corresponding to a third-degree relative). To detect population stratification, principal component analysis was performed using EIGENSOFT 7.2.1 [28]. Three outliers for the genetic population were defined as individuals who had differences in any of the top 10 principal component (PC) scores from the population mean by more than 6 standard deviations. A total of 825 genetically unrelated individuals and 599,876 SNVs passed the quality criteria and were used for subsequent analysis.

Genotype imputation was performed using BEAGLE 4.1 [29], using the 1000 Genomes Project Phase 3 V.5 as the reference panel [30] to infer SNV genotypes that were not directly genotyped by the Japonica Array v2. Imputed SNVs with allelic *R*^2^ < 0.8 or MAF < 1% were filtered out from the imputed data. After the quality control and genotype imputation procedures, 7,080,265 autosomal SNVs in 825 samples were available. To accurately estimate effect sizes of genetic factors associated with SAL, the following individuals were excluded from the study cohort: 4 individuals with a history of pancreatic diseases or chronic kidney disease that could result in SALs above the normal range [5]; 3 individuals with SAL ≥ 200 U/l [5]; 4 individuals with missing values for any of the 5 covariates and SAL. This study cohort for the association analysis consisted of 814 (369 men and 445 women) unrelated individuals.

### Copy number estimation of *AMY1*, *AMY2A*, and *AMY2B* genes

Diploid CNs at the *AMY1*, *AMY2A*, and *AMY2B* loci, respectively, were measured using the QX200 droplet digital PCR (ddPCR) system (Bio-Rad Laboratories Inc., Hercules, CA, USA), following the manufacturer’s instructions and a previously published protocol [13]. The PCR primers and fluorescent probes for three targets (*AMY1*, *AMY2A*, and *AMY2B*) and two references (“Near_AMY” and RPP30) used in the assays are shown in Supplementary Table 1 [13]. The *AMY1* assay employed the PCR primer pairs and probes capable of amplifying and detecting all three genes (*AMY1A*, *AMY1B*, and *AMY1C*) [13]. Target gene probes were labeled with fluorescein amidites (FAM), and control probes with hexachlorofluorescein (HEX).

In brief, genomic DNA was digested with *Hin*dIII restriction enzyme before PCR amplification. Each reaction mixture included 0.45 ng/μl of the digested DNA, 900 nM of each primer, and 250 nM of fluorescently labeled probes (for one target and one reference, respectively) in 20 μl of 1× ddPCR supermix for probes (No UTP; Bio-Rad Laboratories). The “Near_AMY”, just outside the amylase region [13], was used as a reference for the *AMY1* assay, and the *RPP30* gene for *AMY2A* and *AMY2B* assays, respectively. Droplet generation was performed on a QX200 automated droplet generator (Bio-Rad Laboratories), followed by PCR amplification on a Bio-Rad C1000 thermal cycler (Bio-Rad Laboratories). The cycling conditions were as follows: enzyme activation at 95 °C for 10 min; 40 cycles of denaturation at 94 °C for 30 s and annealing/extension at the appropriate temperature (see Supplementary Table 1) for 60 s; and enzyme deactivation at 98 °C for 10 min. The amplified fluorescent droplets were read using a QX200 Droplet Reader (Bio-Rad Laboratories). The droplet counts were analyzed using the QuantaSoft^TM^ software v1.6.6.0320 (Bio-Rad Laboratories) with default settings. The diploid CN of each target gene was estimated from the ratio of the concentration of the target (FAM-labeled) to the concentration of the reference (HEX-labeled), with the diploid CN of the reference being 2. All the estimated values were rounded to the nearest integer values. Seven HapMap sample DNAs (Coriell Cell Repositories, Camden, NJ, USA) with previously known diploid CNs for *AMY1*, *AMY2A*, and *AMY2B* were included as positive controls in each PCR run. Details of the seven HapMap samples used and their estimated diploid CNs [13, 31] are provided in Supplementary Table 2.

### Statistical analysis

To assess associations between SNVs and SAL, multivariate linear regression analysis was performed under an additive genetic model, adjusting for age, sex, BMI, smoking status, drinking habit, and the top 10 PC scores using PLINK 1.9 [32]. The genome-wide significant threshold of the *p* value was set to be 5 × 10^−8^. Manhattan and quantile-quantile plots were generated using the qqman package [33] in R ver.3.6.1 (R Foundation for Statistical Computing, Vienna, Austria). A regional association plot was constructed by LocusZoom (https://my.locuszoom.org) [34] using the 1000 Genomes project Asian (ASN) data (November 2014). Pairwise linkage disequilibrium (LD) measurements between SNVs within SAL-associated genomic regions and association analysis of the SAL-associated SNVs conditioned on the GWAS-identified lead SNV were performed using PLINK 1.9. Significant expression quantitative trait loci (eQTLs) by GWAS SNVs were searched on Genotype-Tissue Expression (GTEx) portal database version 8 (https://gtexportal.org/home/) [35], where the significance was assessed by *p* value and normalized effect size (NES) of the allele on the gene expression. The RegulomeDB (https://regulomedb.org/) was also used to annotate GWAS-identified SNVs with known and predicted regulatory DNA elements such as transcriptional factor–binding sites and DNase hypersensitivity regions [36].

Multivariate linear regression models were used to examine associations between diploid CNVs of *AMY1*, *AMY2A*, and *AMY2B* and SAL, with adjustments for age, sex, BMI, drinking status, smoking habit, and the top 10 PC scores. Per-copy effect size *β* of the respective CNVs and their standard error were estimated in the models using IBM SPSS statistics V25 (SPSS Inc., Chicago, IL, USA). For correlations of diploid CNVs of *AMY1*, *AMY2A*, and *AMY2B* with SAL-associated SNV genotypes, differences in CN between the three SNV genotype groups were tested using the Kruskal–Wallis test with *post hoc* Mann–Whitney U test. To quantify the degree of association between SAL-associated SNVs and *AMY1* diploid CN, a linear regression model was used to estimate the effect size of the lead SNV identified in the GWAS, using the SNV genotype (coded as 0, 1, or 2 based on the number of effect alleles) as a predictor for *AMY1* diploid CN. The independence of *AMY1* CN and SAL-associated SNV was further assessed using a multiple linear regression model adjusting for age, sex, BMI, smoking status, drinking habit, and the top 10 PC scores.

To assess associations of SAL-associated *AMY1* diploid CN and SNVs with BMI, BMI was standardized using a rank-based inverse-normal transformation in a linear regression model adjusted for age, age-squared, sex, and the top 10 PC scores as covariates [34]. The linear regression analysis was performed using PLINK 1.9 and SPSS statistics V25. In the association study for BMI, SNV rs11642015 in the *FTO* gene, which has had the largest effect size on BMI in the Japanese population [37], was also analyzed. *P* values < 0.05 were considered statistically significant. All the figures were drawn in the R environment (version 3.6.2).

## Results

### SNV-based GWAS for SAL

**Fig. 1:**
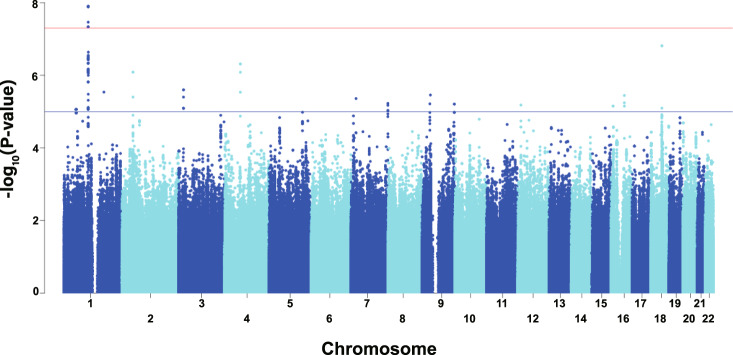
Manhattan plot of associations from GWAS for serum amylase levels (SALs). The -log_10_-transformed *p* values (*y*-axis) of all the analyzed autosomal SNVs are plotted against their physical positions along the chromosome (*x*-axis). The horizontal red line represents the genome-wide significance threshold (*p* = 5 × 10^−8^)

**Fig. 2:**
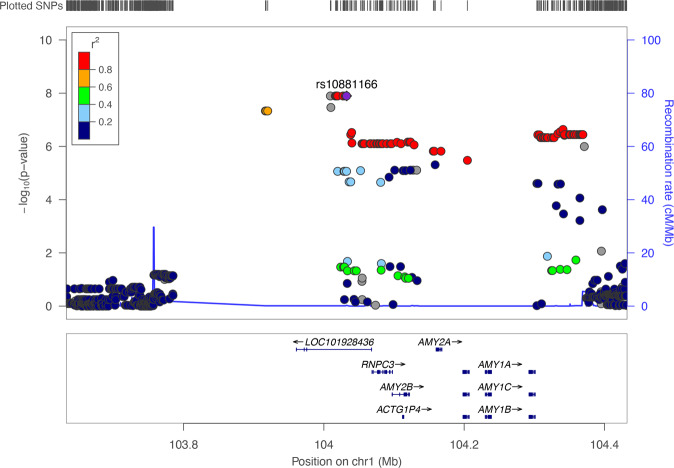
Regional association plot for the SAL-associated locus on chromosome 1. The left and right Y-axes represent -log_10_-transformed *p* values of SNVs and the recombination rates along the chromosome, respectively. Purple diamond and circles represent the GWAS-lead SNV rs10881166 and other SNVs within the region, respectively; the color is based on the degree of linkage disequilibrium (pairwise *r*^2^) with rs10881166 using the 1000 Genomes project Asian (ASN) data (November 2014). The RefSeq genes within the region are shown in the panel below

To identify the loci associated with interindividual differences in SALs, we conducted a GWAS for SAL using 814 genetically unrelated participants and 7,080,265 autosomal SNVs. The baseline characteristics of the participants and associations between SAL and the 5 demographic and lifestyle covariates used are shown in Table 1. The Manhattan and quantile-quantile plots of the GWAS are shown in Fig. 1 and Supplementary Fig. 1, respectively. As shown in Table 2 and Fig. 2, we found 14 SNVs showing genome-wide significant associations for SAL within a genomic region in and near the *LOC101928436* gene (current official symbol, *RNPC3-DT*) on chromosome 1. The SAL-associated region was also located ~60 kb upstream of amylase gene cluster (Fig. 2).

**Table 1:** Baseline characteristics of subjects participating in the GWAS, and univariate analysis of associations of serum amylase levels with the 5 demographic and lifestyle covariates used

	GWAS cohort	Univariate analysis		
		*β*	SE	*p* value
*N*	814	–	–	–
Serum amylase (U/l), mean ± SD	82.2 ± 26.0	–	–	–
Age (years), mean ± SD	61.7 ± 10.8	0.29	0.08	0.001
Female, *n* (%)	445 (54.7)	−1.22	1.84	0.50
BMI (kg/m^2^), mean ± SD	23.3 ± 3.3	−1.51	0.28	5.43 × 10^−8^
Current smoker, *n* (%)	148 (18.2)	−10.30	2.34	1 × 10^−5^
Drinker, *n* (%)	395 (48.5)	−7.54	1.81	4 × 10^−5^

*β* regression coefficient, *SE* standard error of the regression coefficient, *SD* standard deviation, *BMI* body mass index

**Table 2:** 14 top-ranked SNVs (*p* < 5 × 10^−8^) in GWAS for SAL

SNV ID	CHR	Position (hg19)	Nearest gene	EA/Non-EA	EAF	*β*	SE	*p* value
rs10785777	1	103916755	*LOC101928436*	C/T	0.39	−7.00	1.27	4.61 × 10^−8^
rs4285741	1	103917589	*LOC101928436*	T/C	0.39	−7.00	1.27	4.61 × 10^−8^
rs10785846	1	103920016	*LOC101928436*	G/A	0.39	−7.00	1.27	4.61 × 10^−8^
rs10629010	1	104009348	*LOC101928436*	CTAT/C	0.34	−7.36	1.28	1.27 × 10^−8^
rs10712365	1	104010119	*LOC101928436*	T/TA	0.34	−7.13	1.28	3.43 × 10^−8^
rs7417147	1	104016534	*LOC101928436*	A/G	0.34	−7.36	1.28	1.27 × 10^−8^
rs6696797	1	104017778	*LOC101928436*	A/G	0.34	−7.36	1.28	1.27 × 10^−8^
rs6663215	1	104018310	*LOC101928436*	G/A	0.34	−7.36	1.28	1.27 × 10^−8^
rs7553516	1	104019702	*LOC101928436*	T/G	0.34	−7.36	1.28	1.27 × 10^−8^
rs12266225	1	104026573	*LOC101928436*	A/G	0.34	−7.36	1.28	1.27 × 10^−8^
rs12945461	1	104027213	*LOC101928436*	G/A	0.34	−7.36	1.28	1.27 × 10^−8^
rs75729251	1	104027277	*LOC101928436*	A/G	0.34	−7.36	1.28	1.27 × 10^−8^
rs10158225	1	104030621	*LOC101928436*	C/T	0.34	−7.36	1.28	1.27 × 10^−8^
rs10881166	1	104032552	*LOC101928436*	A/C	0.34	−7.36	1.28	1.27 × 10^−8^

Data are shown as the per-allele regression coefficient *β*, SE, and *p* value using an additive genetic model with adjustments for age, sex, BMI, smoking status, drinking habit, and the top 10 principal component scores

*SNV* single-nucleotide variant, *CHR* chromosome, *EA* effect allele, *EAF* effect allele frequency, *β* regression coefficient of effect allele, *SE* standard error of the regression coefficient

Ten of the 14 GWAS-identified SNVs had the same effect sizes on SAL (per-allele *β* = −7.36, *p* = 1.27 × 10^−8^) and were in complete LD (*D*′ = 1, *r*^2^ = 1) with each other, indicating that these SNVs are equivalent as GWAS signals. Among the 10 SNVs, we selected rs10881166, the SNV closest to the amylase gene cluster, as the lead SNV for the subsequent association analyses. A representative additive effect of the allele dosage of rs10881166 on SAL is shown in Supplementary Fig. 2. Functional annotation of the 10 top-ranked SNVs showed that rs10881166 was a significant eQTL of *AMY2B* in the brain putamen tissues (NES = −0.19, *p* = 1.7 × 10^−7^), similar to other SNVs examined, according to the GTEx portal database (Supplementary Table 3). The effect allele (A) of rs10881166 was associated with low *AMY2B* expression in the brain tissues (Supplementary Fig. 3). Based on the RegulomeDB, rs10881166 showed the lowest RegulomeDB rank (3a) among the 10 top-ranked SNVs, predicting that it may be involved in regulatory functions of gene expression (Supplementary Table 3).

To determine the number of independent association signals within the SAL-associated genomic region, we examined the association of every SNV within the genomic interval and SAL, adjusting for the effect of the lead SNV rs10881166. This conditioned analysis showed that none of the SNVs examined were significantly associated with SAL (Supplementary Table 4), indicating that there was likely one association signal within the SAL-associated genomic region.

### Association analysis of *AMY1*, *AMY2A*, and *AMY2B* CNs with SAL

**Fig. 3:**
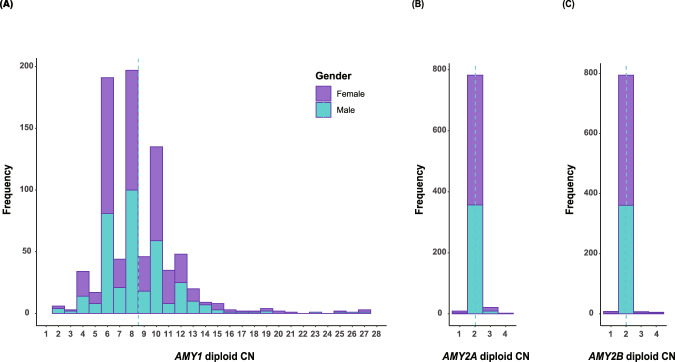
Histograms of estimated diploid CN distributions of (**A**) *AMY1*, (**B**) *AMY2A*, and (**C**) *AMY2B* genes in the studied population. Male and female participants are shown in green and purple, respectively. The dotted lines in the respective panels represent the mean diploid CN values by sex

As shown in Fig. 3, in our study cohort, the diploid CNs of the *AMY1* genes (comprising *AMY1A*, *AMY1B*, and *AMY1C*) ranged from 2 to 27, and those of *AMY2A* and *AMY2B*, from 1 to 4 copies. The average number of diploid CNs in the analyzed population was 8.5 for *AMY1*, and 2 for *AMY2A* and *AMY2B* (Table 3). Similar to the previous study [13], we found that those with even diploid CNs of *AMY1* (e.g., CN = 4, 6, 8, 10, 12) are more dominant than those with odd ones. Individuals with extremely high CNs of *AMY1* (more than 20 diploid CNs) were found at a frequency of ~1%. Most individuals had 2 diploid CNs for *AMY2A* and *AMY2B* (96% and 98% of the population, respectively). There were no significant differences in the mean diploid CN at each of the three loci between the sexes.

**Table 3:** Associations between *AMY1*, *AMY2A*, and *AMY2B* gene copy numbers and SAL

Diploid CN	Mean ± SD	Multivariate analysis		
		*β*	SE	*p* value
*AMY1* CN	8.5 ± 3.2	2.44	0.26	1.89 × 10^−19^
*AMY2A* CN	2.0 ± 0.23	2.39	3.85	0.54
*AMY2B* CN	2.0 ± 0.22	5.81	4.01	0.15

Data are shown as the per-copy regression coefficient *β*, SE and *p* value, adjusted for age, sex, BMI, smoking status, drinking habit and the top 10 principal component scores

*SD* standard deviation, *β* regression coefficient of the gene copy, *SE* standard error of the regression coefficient

The multivariate linear regression models revealed a significant positive association between *AMY1* CN and SAL (per-copy *β* = 2.44, *p* = 1.89 × 10^−19^) (Table 3). In contrast, no significant association with SAL was found for *AMY2A* CN (*p* = 0.54) or *AMY2B* CN (*p* = 0.15) (Table 3).

### Relative impacts of GWAS-identified SNV and *AMY1* CN on SAL

LD estimation between GWAS-identified SNVs and amylase CNVs was not possible because the ddPCR method provided the total copy number per individual (diploid CN), not the genotype of the individual in copy number. To infer the degree of association between the SAL-associated GWAS signal near amylase gene cluster and amylase gene CNVs, we examined the relationship between the genotypes of the lead SNV rs10881166 and diploid CNs of AMY in our cohort. There were significant differences in the diploid CNs of *AMY1* among the three genotype groups of rs10881166 (*p* = 3.52 × 10^−56^), but not in the diploid CNs of *AMY2A* (*p* = 0.64) and *AMY2B* (*p* = 0.73) (Supplementary Fig. 4). Linear regression analysis showed that the effect allele (A) of rs10881166 was significantly associated with a 2.2 CN decrease in *AMY1* diploid CN (*p* = 2.23 × 10^−46^, Pearson’s correlation *r* = 0.47) (Supplementary Table 5), indicating that the GWAS-identified SNV explains some of the variation in *AMY1* diploid CN.

To determine the relative contribution of the lead SNV and *AMY1* CNV to variation in SALs, we performed a multivariate linear regression analysis, adjusted for age, sex, BMI, drinking status, smoking habit, and the top 10 PC scores. In the model, *AMY1* CN showed a significant association with SAL (per-copy *β* = 2.18, *p* = 5.4 × 10^–13^), while the lead SNV rs10881166 was not significantly associated with SAL (per-allele *β* = −2.50, *p* = 0.08). These results suggest that the SAL-associated lead SNVs are partially genetically correlated with *AMY1* CNV.

### Association analysis of *AMY1* CN and GWAS-identified SNV with BMI

Similar to previous studies in the Japanese population [5, 6], we found a significant negative correlation between SAL and BMI (*β* = −1.51, *p* = 5.43 × 10^−8^) (Table 1). Although the amylase locus has not been identified in GWASs for obesity or BMI, the association of *AMY1* CNV with obesity or BMI has been noteworthy [13, 19]. Therefore, we further evaluated the association of *AMY1* CN and GWAS-identified SNV with BMI using the SHIP cohort. First, we confirmed that the *FTO* SNV rs11642015, which has been associated with BMI in the Japanese population [37], was significantly associated with BMI (per-allele *β* = 0.13, *p* = 0.03) at a nominal significant level in the study cohort (Supplementary Table 6). Furthermore, we found that neither *AMY1* CN (per-copy *β* = −0.02, *p* = 0.11) nor SAL-associated SNV rs10881166 (per-allele *β* = 0.07, *p* = 0.16) was significantly associated with BMI (Supplementary Table 6), suggesting that SAL-associated *AMY1* CNV and SNVs are unremarkable for BMI in the Japanese population.

## Discussion

Recently, studies attempting to identify protein quantitative trait loci (pQTL) for the human blood proteome have received considerable attention [38–41]. The present population-based study was conducted to characterize the genetic architecture of SAL in Japanese individuals. In GWAS with autosomal SNVs after adjustments for the effects of non-genetic factors on SAL, we found a SAL-associated locus near the amylase gene cluster on chromosome 1. Diploid CN association analysis showed a significant association with SAL in *AMY1* but not in *AMY2A* and *AMY2B*. Joint association analysis using GWAS-lead SNV and *AMY1* diploid CN revealed that *AMY1* CN was the primary genetic factor to control individual differences in SAL in the Japanese population.

Many observational studies have suggested that amylase is associated with susceptibility to complex traits and diseases of humans such as obesity and insulin resistance [12, 15, 19, 22–24], but their causal relationship remains unclear. Studying the genetic factors that explain individual differences in human amylase levels would provide a better understanding of the associations between amylase and disease risks. To the best of our knowledge, this is the first study to map significant pQTL for SAL in a Japanese population. The findings of this study are consistent with previous GWAS reports that pQTLs for blood AMY1A and AMY2B proteins in European-descent populations are located within or near the genes encoding amylase [38, 39]. Although the causative variant at this locus that affects blood amylase levels and the mechanism of action are unknown, the present association analysis reveals that the contribution of *AMY1* CNV to individual differences in SAL is higher than that of other common variants, including SNVs. This suggests a dosage-dependent effect of the *AMY1* gene on SAL in the Japanese population.

In addition to the complex genomic structure of the amylase locus, including duplications, inversions, and deletions [11, 31], *AMY1* diploid CNs are multiallelic and very diverse in the population, making it difficult to find the best-tagging SNV or haplotype that predicts the diploid or haploid CN of each individual with high accuracy [13]. Therefore, the diploid CNs of the participants were experimentally determined in this study using the ddPCR method. Because the ddPCR method did not allow genotyping of participants with respect to amylase CN, it was not possible to directly measure the degree of LD between *AMY1* CNV and SNVs. Usher et al. have reported that SNVs near the AMY were in weak LD (*r*^2^ < 0.3) with the respective amylase structural haplotypes with different numbers of AMY (*AMY1*, *AMY2A*, and *AMY2B*) [13]. Based on the linear regression analysis between variants in this study, the significant correlation between the *AMY1* diploid CNs and genotypes of GWAS-lead SNV may be attributed to weak LD between the two variants, which may have attenuated the impact of the GWAS-lead SNV on SAL in the joint association analysis using GWAS-lead SNV and *AMY1* diploid CN. This indicates that the association between the GWAS-identified SNVs and SAL may be mediated, at least in part, by the dosage-dependent effect of the *AMY1* gene on SAL. On the other hand, the GWAS-lead SNV is an eQTL for *AMY2B* in the GTEx portal database. To the best of our knowledge, there is no evidence that the expression levels of *AMY2B* vary by *AMY1* CNV; therefore, we cannot rule out the possibility that the GWAS-lead SNV may also contribute to individual differences in SAL via the genotype-dependent changes in *AMY2B* gene expression. A deeper understanding of the detailed genomic structure of the amylase locus, the haplotype structures consisting of SNVs and CNVs, and the functional elements involved in amylase gene expression within the locus will allow us to fully elucidate the impact of the amylase locus on human SAL.

One of the implications of our study was to assess the association of SAL and SAL-associated genetic factors such as *AMY1* diploid CNs with BMI. This population-based study showed a significant negative association between SAL and BMI but no association of *AMY1* diploid CNs and GWAS-lead SNV with BMI. Although findings regarding the association of *AMY1* CNV with BMI or obesity have been inconsistent in populations worldwide [13, 19–21], this study joins a list of studies that have demonstrated that *AMY1* diploid CN is not associated with BMI. Given that no BMI-associated signals within or near the amylase locus have been found in previous GWASs for BMI using SNVs in East Asian populations, including Japanese populations [37, 42], it seems unlikely that salivary amylase *AMY1* CNV has a large enough effect on BMI in the Japanese population. To conclude whether *AMY1* CNV and SAL-associated SNVs are associated with BMI, association analyses would need to be conducted using large cohorts with sufficient statistical power after adjusting for the effects of non-genetic factors (e.g., daily energy intake, exercise frequency) that can influence BMI variation.

The current GWAS with autosomal SNVs shows no significant associations of SNVs other than the amylase locus with SAL in the Japanese population, probably due to the limited statistical power to identify common SNVs associated with SAL. To gain a better understanding of the polygenic nature of this trait in humans, future large-scale association analyses using whole-genome sequence data from diverse populations that can analyze all types of variants, including rare variants, are required. In addition, it is also necessary to perform amylase isozyme-specific association analyses because salivary and pancreatic types, which constitute serum amylase, are thought to be separately regulated for gene expression. The findings from such future studies using sophisticated genetic analyses such as Mendelian randomization analysis will allow us to assess whether amylase has causal roles in human diseases and traits.

## Supplementary information

Supplementary Table 1

Supplementary Table 2

Supplementary Table 3

Supplementary Table 4

Supplementary Table 5

Supplementary Table 6

Supplementary Figure 1

Supplementary Figure 2

Supplementary Figure 3

Supplementary Figure 4
