# Supplementary material for: Genetic factors associated with serum amylase in a Japanese population: combined analysis of copy-number and single-nucleotide variants
Source: J Hum Genet. 2023 Jan 4;68(5):313–9. doi: 10.1038/s10038-022-01111-3 (PMC10125868; doi:10.1038/s10038-022-01111-3)
Supplement: Supplementary file 1 — Supplementary Table 1 [file 10038_2022_1111_MOESM1_ESM.docx]

**Supplementary Table 1. ddPCR primers and probes**

| Assay name (target/reference) | Forward primer^1^ | Reverse primer^1^ | Probe^1^ | Annealing/extension temperature |
| --- | --- | --- | --- | --- |
| AMY1_assay1 (*AMY1*) | AAAAACCCAAGAATTAGGAATGG | CCTGGAAGGATTTTCTGGTG | TGCTCTCATTTTTAGATGACTTGTG | 54.1°C |
| AMY2A_assay1 (*AMY2A*) | AACATCAAAAAGTCTCTCATGGAA | CAAATTTTGGTTTTCTACTGTTATGTG | GGCCCCAGCAACAGGTCACTG | 56°C |
| AMY2B_assay1 (*AMY2B*) | GGATGCGATCATCTTGATCTT | CCCAGACCATCTTCAACTCC | GTAGGCCAACACGGTGCTAT | 56°C |
| Near_AMY (reference; *AMY1* assay) | AAATTTATTGGAGGGAT GTTGG | TTCAAGTTTGACTGCTAAC TCCTG | TGGAATAAAGAATCATTGGGCACAGGT | 54.1°C |
| RPP30 (reference; *AMY2A* and *AMY2B* assays) | GATTTGGACCTGCGAGCG | GCGGCTGTCTCCACAAGT | CTGACCTGAAGGCTCT | 56°C |

^1^ obtained from Usher et al. (13).
