# Supplementary material for: Genetic factors associated with serum amylase in a Japanese population: combined analysis of copy-number and single-nucleotide variants
Source: J Hum Genet. 2023 Jan 4;68(5):313–9. doi: 10.1038/s10038-022-01111-3 (PMC10125868; doi:10.1038/s10038-022-01111-3)
Supplement: Supplementary file 2 — Supplementary Table 2 [file 10038_2022_1111_MOESM2_ESM.docx]

**Supplementary Table 2. Pre-identified CNs for *AMY1*, *AMY2A*, and *AMY2B* in seven HapMap samples**

| Sample ID | Population | Estimated diploid CNs^1^ | | |
| --- | --- | --- | --- | --- |
|  |  | *AMY1* | *AMY2A* | *AMY2B* |
| NA18961 | JPT | 6 | 2 | 2 |
| NA19119 | YRI | 12 | 4 | 2 |
| NA12813 | CEU | 4 | 0 | 2 |
| NA19099 | YRI | 3 | 3 | 3 |
| NA12763 | CEU | 7 | 3 | 3 |
| NA18998 | JPT | 10 | 2 | 2 |
| NA11829 | CEU | 5 | 1 | 2 |

^1^ Based on previous findings from Usher et al. (13) and Carpenter et al. (31).

CN, copy number; JPT, Japanese in Tokyo; CEU, Utah residents with Northern and Western European ancestry from the CEPH collection; YRI, Yoruba in Ibadan, Nigeria.
