# Supplementary material for: Genetic factors associated with serum amylase in a Japanese population: combined analysis of copy-number and single-nucleotide variants
Source: J Hum Genet. 2023 Jan 4;68(5):313–9. doi: 10.1038/s10038-022-01111-3 (PMC10125868; doi:10.1038/s10038-022-01111-3)
Supplement: Supplementary file 3 — Supplementary Table 3 [file 10038_2022_1111_MOESM3_ESM.docx]

**Supplementary Table 3. Functional annotation of 10 top-ranked GWAS-identified SNVs**

| SNV | CHR | Position (hg19) | EA/Non-EA | GTEx portal database V8 | | | RegulomeDB |
| --- | --- | --- | --- | --- | --- | --- | --- |
|  |  |  |  | eQTL Gene | *p*-value | NES | Rank |
| rs10629010 | 1 | 104009348 | CTAT/C | *AMY2B* | 6.40 × 10^-8^ | -0.19 | 6 |
| rs7417147 | 1 | 104016534 | A/G | *AMY2B* | 1.70 × 10^-7^ | -0.19 | 7 |
| rs6696797 | 1 | 104017778 | A/G | *AMY2B* | 1.70 × 10^-7^ | -0.19 | 4 |
| rs6663215 | 1 | 104018310 | G/A | *AMY2B* | 1.70 × 10^-7^ | -0.19 | 5 |
| rs7553516 | 1 | 104019702 | T/G | *AMY2B* | 1.70 × 10^-7^ | -0.19 | 5 |
| rs12266225 | 1 | 104026573 | A/G | N/A | N/A | N/A | N/A |
| rs12945461 | 1 | 104027213 | G/A | *AMY2B* | 1.70 × 10^-7^ | -0.19 | 6 |
| rs75729251 | 1 | 104027277 | A/G | *AMY2B* | 1.70 × 10^-7^ | -0.19 | 7 |
| rs10158225 | 1 | 104030621 | C/T | *AMY2B* | 1.70 × 10^-7^ | -0.19 | 7 |
| rs10881166 | 1 | 104032552 | A/C | *AMY2B* | 1.70 × 10^-7^ | -0.19 | 3a |

The RegulomeDB ranks (https://regulomedb.org/) are categorical scores that annotate the functional impacts of SNVs based on known and predicted regulatory DNA elements, ranging from 1a to 7, with lower ranks indicating a greater likelihood that the SNV has a regulatory function.

eQTL, expression quantitative trait loci; NES, normalized effect size; N/A, not applicable.
