# Supplementary material for: Genetic factors associated with serum amylase in a Japanese population: combined analysis of copy-number and single-nucleotide variants
Source: J Hum Genet. 2023 Jan 4;68(5):313–9. doi: 10.1038/s10038-022-01111-3 (PMC10125868; doi:10.1038/s10038-022-01111-3)
Supplement: Supplementary file 4 — Supplementary Table 4 [file 10038_2022_1111_MOESM4_ESM.docx]

**Supplementary Table 4. Conditional association analysis of GWAS-identified SNVs with SAL by adjusting for the effect of the lead SNV rs10881166**

| SNV ID | CHR | Position (hg19) | EA/Non-EA | Pairwise LD (*r*^2^-value) | *β* | SE | *p*-value |
| --- | --- | --- | --- | --- | --- | --- | --- |
| rs10785777 | 1 | 103916755 | C/T | 0.74 | -2.84 | 2.48 | 0.25 |
| rs4285741 | 1 | 103917589 | T/C | 0.74 | -2.84 | 2.48 | 0.25 |
| rs10785846 | 1 | 103920016 | G/A | 0.74 | -2.84 | 2.48 | 0.25 |
| rs10712365 | 1 | 104010119 | T/TA | 0.91 | -1.49 | 4.13 | 0.71 |

Among the GWAS-identified SNVs, 9 SNVs that are in complete LD with the lead SNV rs10881166 were not analyzed because of multicollinearity. Pairwise LD measures between the lead SNV and each of the analyzed SNVs are based on *r*^2^-values. Data are shown as the per-allele regression coefficient *β*, SE, and *p*-value using an additive genetic model after adjusting for the effects of the conditioned SNV (rs10881166), age, sex, BMI, smoking status, drinking habit, and the top 10 principal component scores.

SNV, single nucleotide variant; CHR, chromosome; EA, effect allele; *β*, regression coefficient of effect allele; SE, standard error of the regression coefficient.
