# Supplementary material for: Genetic factors associated with serum amylase in a Japanese population: combined analysis of copy-number and single-nucleotide variants
Source: J Hum Genet. 2023 Jan 4;68(5):313–9. doi: 10.1038/s10038-022-01111-3 (PMC10125868; doi:10.1038/s10038-022-01111-3)
Supplement: Supplementary file 5 — Supplementary Table 5 [file 10038_2022_1111_MOESM5_ESM.docx]

**Supplementary Table 5. Association of rs10881166 with *AMY1* diploid CN**

| SNV ID | EA/Non-EA | *AMY1* copy number association | | | |
| --- | --- | --- | --- | --- | --- |
|  |  | *β* | SE | *p-*value | Pearson’s correlation *r* |
| rs10881166 | A/C | -2.2 | 0.14 | 2.23 × 10^-46^ | 0.47 |

SNV genotype was used as a coded predictor variable, 0, 1, or 2, based on the number of the effect alleles.

SNV, single nucleotide variant; EA, effect allele; *β*, regression coefficient of effect allele; SE, standard error of the regression coefficient.
