# Supplementary material for: Genetic factors associated with serum amylase in a Japanese population: combined analysis of copy-number and single-nucleotide variants
Source: J Hum Genet. 2023 Jan 4;68(5):313–9. doi: 10.1038/s10038-022-01111-3 (PMC10125868; doi:10.1038/s10038-022-01111-3)
Supplement: Supplementary file 6 — Supplementary Table 6 [file 10038_2022_1111_MOESM6_ESM.docx]

**Supplementary Table 6. Association analyses of *AMY1* CN, SAL-associated SNV, and previously known BMI-associated SNV with BMI**

| Variant | Nearest gene | EA/Non-EA | *β* (per-copy or per-allele) | SE | *p*-value |
| --- | --- | --- | --- | --- | --- |
| Diploid copy number | *AMY1* | – | -0.02 | 0.01 | 0.11 |
| rs10881166 | *LOC101928436* | A/C | 0.07 | 0.05 | 0.16 |
| rs11642015 | *FTO* | T/C | 0.13 | 0.06 | 0.03 |

In the linear regression analysis, BMI was standardized using a rank-based inverse-normal transformation in a linear regression model adjusted for age, age-squared, sex, and the top 10 PC scores as covariates [37].

EA, effect allele; *β*, regression coefficient of *AMY1* copy number or effect allele; SE, standard error of the regression coefficient.
